# A hybrid attention-enhanced DenseNet neural network model based on improved U-Net for rice leaf disease identification

**DOI:** 10.3389/fpls.2022.922809

**Published:** 2022-10-18

**Authors:** Wufeng Liu, Liang Yu, Jiaxin Luo

**Affiliations:** ^1^ School of Artificial Intelligence and Big Data, Henan University of Technology, Zhengzhou, China; ^2^ College of Electrical Engineering, Henan University of Technology, Zhengzhou, China

**Keywords:** rice leaf disease identification, densenet, U-net, deep learning, convolution networks, ROI extraction

## Abstract

Rice is a necessity for billions of people in the world, and rice disease control has been a major focus of research in the agricultural field. In this study, a new attention-enhanced DenseNet neural network model is proposed, which includes a lesion feature extractor by region of interest (ROI) extraction algorithm and a DenseNet classification model for accurate recognition of lesion feature extraction maps. It was found that the ROI extraction algorithm can highlight the lesion area of rice leaves, which makes the neural network classification model pay more attention to the lesion area. Compared with a single rice disease classification model, the classification model combined with the ROI extraction algorithm can improve the recognition accuracy of rice leaf disease identification, and the proposed model can achieve an accuracy of 96% for rice leaf disease identification.

## Introduction

The pressure on the agriculture sector will increase with the continuing expansion of the human population and so agri-technology and precision farming have gained much importance in today’s world (Jha et al., 2019). The digital transformation of agriculture has evolved various aspects of management into artificial intelligent systems for the sake of making value from the ever-increasing data originated from numerous sources (Benos et al., 2021). Rice is one of the most widely consumed grains in the world. As the most populous country in the world, China also consumes more rice than any other country, with about 154.9 million metric tons consumed in 2021/2022. Rice leaf diseases directly affect the quality and yield of rice (Yang et al., 2021). Brown spot, Bacterial blight, and Leaf blast are three kinds of the most prevalent rice plant diseases (Azim et al., 2021). Therefore, the classification and control of leaf diseases are crucial in rice cultivation. Traditional disease identification mainly relies on manual observation and identification (Dutot et al., 2013), which is labor-intensive and requires extensive experience to identify accurately.

With the rapid advance of deep learning techniques in the field of computer vision, image-based crop disease identification has attracted much attention. Researchers have thus attempted to automate the process of plant disease detection and classification using leaf images (Kaur et al., 2019). The deep convolutional neural network (DCNN), which excels in image classification and detection, is widely used in crop disease identification (Krizhevsky et al., 2017). Xie et al. (2020) designed an improved convolutional neural network to achieve effective identification of grape leaf diseases, and the mAP reached 81.1%. Jiang et al. (2019) achieved successful recognition of apple leaf diseases by improving the convolutional neural networks model, and the mAP reached 78.8%. Chen et al. (2020) achieved an average accuracy of 92.00% for rice plant image class prediction using the transfer learning method. Goluguri et al. (2021) proposed technique EAFSO associates DCNN-LSTM identifies the rice diseases with 97.5% accuracy. Jiang et al. (2021) realized the identification of rice leaf diseases and wheat leaf diseases through the method of migration learning, and their identification accuracy of rice leaf diseases reached 97.22%, and the identification accuracy of wheat leaf diseases reached 98.75%. The Elliptical-Maximum Margin Criterion metric learning was used to study the identification of wheat leaf disease image. The wheat image was segmented through the Ostu method, achieving 94.16% accuracy of the identification of wheat diseases (Bao et al., 2021). Machine learning methods were used to classify the hyperspectral images of grape leaves to identify grapevine leaf roll disease. The highest identification accuracy reached 89.93%, which meant that machine learning methods could effectively detect grapevine leaf diseases during asymptomatic stages (Gao et al., 2020). The deep learning network of migration learning was used to conduct image identification research on four kinds of camellia diseases, they used the AlexNet model to pre-train on ImageNet, and designed a new fully connected layer, then got a mean validation accuracy of 91.25% (Long et al., 2018). Since there are various types of rice leaf diseases and the lesion characteristics exhibited by similar diseases vary greatly, it is difficult to rely on the basic neural network model to classify and identify rice leaf diseases, and the results are not ideal (Feng et al., 2021). Sharma et al. (2022) used transfer learning techniques to identify three rice diseases: bacterial blight, rice blast, and brown spot, with 99.5% accuracy. Neural network technology has made its mark in the field of crop disease identification. It not only improves the accuracy of crop disease identification, but also saves time and labor cost. The novelty of this study is using an improved U-Net for ROI extraction. The ROI extraction allows the computer to automatically select out the region of interest and identify the most appropriate result or the best image that provides more information than other images. There are many applications of ROI in the field of computer vision, as shown in **Table 1**.

**Table 1:** The related works of ROI.

Researchers	Method	Objectives	Performance
Hoang et al. (2019)	Based on the adaptive ROI and deep CNN	Detection and classification of road markings	Precision:0.997 Recall:0.972 Accuracy:0.969 F1_score:0.984
Mitra et al. (2018)	CNN	Retinal fundus image analysis without manual intervention	The method accomplish an accuracy of 99.05% and 98.78% on the Kaggle and MESSIDOR test sets for ROI detection
Ashraf et al. (2020)	Improved k-mean algorithm	Detection of melanoma	The proposed system gives 97.9% and 97.4% accuracy for DermIS and DermQuest respectively
Yap et al. (2020)	Faster-RCNN with Inception-ResNet-v2	Breast ultrasound ROI detection and lesion localisation	IoU:0.8535 Recall:0.9358 Precision:0.9003 F1_score:0.9080 FPI:0.0982

After pre-processing of healthy and diseased (Brown spot, Leaf blight, Bacterial blast) rice leaf images from Kaggle, the present study uses ROI extraction to identify and extract lesion areas. Then disease area images were put into a hybrid attention-enhanced DenseNet model based on improved U-Net for accurate identification of disease types. This study uses the Dice coefficient, Accuracy, Precision, Recall, F1-Score, AUC, and confusion_matrix as model evaluation criteria. The experiments show that under the condition of a small dataset, the accuracy of the classification after ROI extraction was significantly higher than those without it, and the accuracy of the validation dataset could reach 96%, which meets the requirements of rice leaf disease identification and classification.

## Materials and equipment

**Figure 1:**
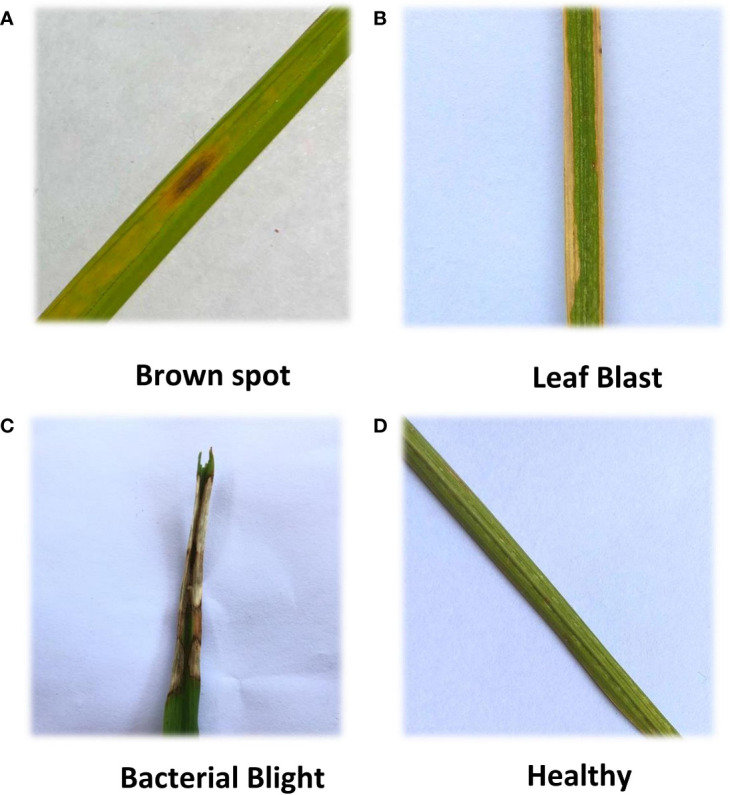
Four kinds of rice leaf images. **(A)** Brown spot, **(B)** Leaf Blast, **(C)** Bacterial Blight, **(D)** Healthy. Four kinds of rice leaf images showing different morphology.

Four types of original rice leaf images from Kaggle. Healthy, Brown spot, Leaf Blast, and Bacterial Blight were collected as the experimental dataset. The training sample collection method is a separate shooting after collecting rice leaf samples in the field. To ensure a balanced distribution of the dataset, some training samples were generated using the Gan network and added to the dataset. These images are stored in PNG format. The four kinds of rice leaf images are shown in **Figure 1**.

**Table 2** lists a total of 2988 images of rice leaves in four categories including Brown spot, Leaf Blast, Bacterial Blight, and Healthy. Of these, 540 were Brown spot, 556 were Leaf Blast, 404 were Bacterial Blight, and 1488 were Healthy.

**Table 2:** Distribution of rice leaf dataset.

Leaf type	Total
Brown spot	540
Leaf Blast	556
Bacterial Blight	404
Healthy	1488

**Figure 2:**
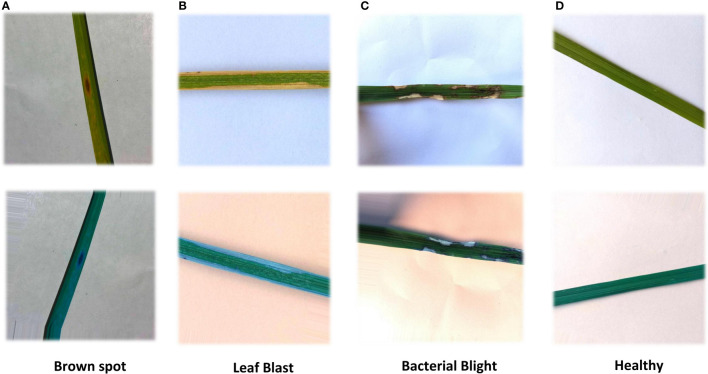
Rice images with random image enhancement. The enhancement includes horizontal flip, vertical flip, scaling horizontal offset, vertical offset, and shear transform. **(A)** Brown spot **(B)** Leaf Blast, **(C)** Bacterial Blight, **(D)** Healthy.

**Figure 3:**
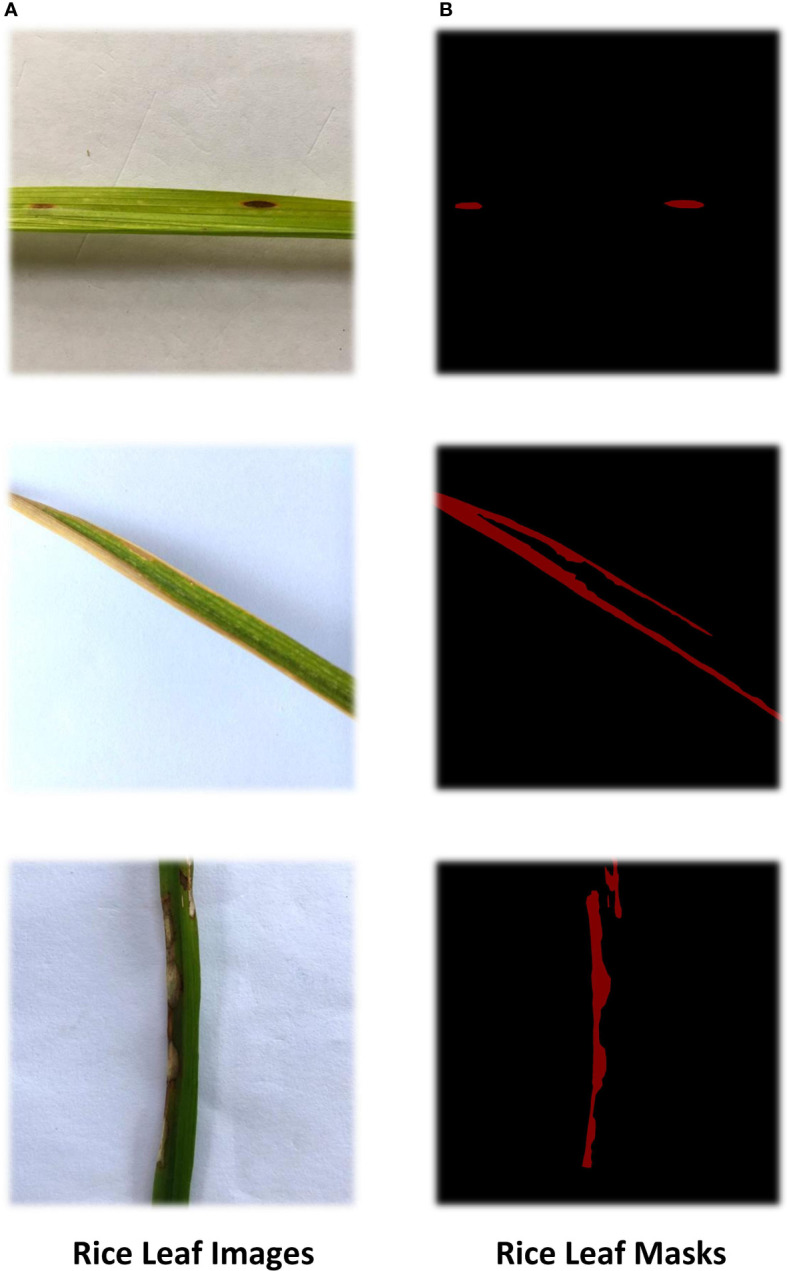
Masks of rice leaf. Rice leaf images were labeled by Labelme to generate masks.The original pictures are shown in **(A)**, the red areas in **(B)** is the diseased areas.

As the resolution ratio of collected rice leaf images was high and different, direct training would greatly increase the computational load of the model and lead to long training time, so images were cut into 256×256. Considering that the increased amount of data could improve the training effectiveness of the deep neural network model, the samples were all treated with random image enhancement, including random rotations, random zoom, random shifts, and shear transformation. **Table 3** shows augmentation parameters. The results are shown in **Figure 2**. In addition, the disease areas were annotated by Labelme to get the masks used for the ROI extraction model training. This is shown in **Figure 3**. There are 540 masks for Brown spot, 549 masks for Leaf Blast, and 404 masks for Bacterial Blight, for a total of 1500 masks. Healthy images will not be annotated and blank masks will be output directly before the model is trained. The train and test datasets were divided according to a ratio of 0.7:0.3, with 2092 samples in the training dataset and 896 samples in the test dataset.

**Table 3:** Parameters of the augmentation techniques applied in the current study.

Augmentation technique	Parameter with value
Random Rotations	Rotation_range = 30
Random Zoom	Zoom_range = 0.2
Random Shifts	Width_shift_range = 0.1 Height_shift-range = 0.2
Shear Transformation	Shear_range = 0.2

Deep learning was conducted using Python 3.8 with a Tensorflow 2.4 framework with GPU acceleration. A computer with AMD Ryzen 5 5600x CPU, 16 GB of RAM, an NVidia GeForce RTX3070 GPU (8GB RAM, CUDA cores 5888, CUDA version 11.1), and a 256GB SSD were used for calculation.

## Methods

**Figure 4:**
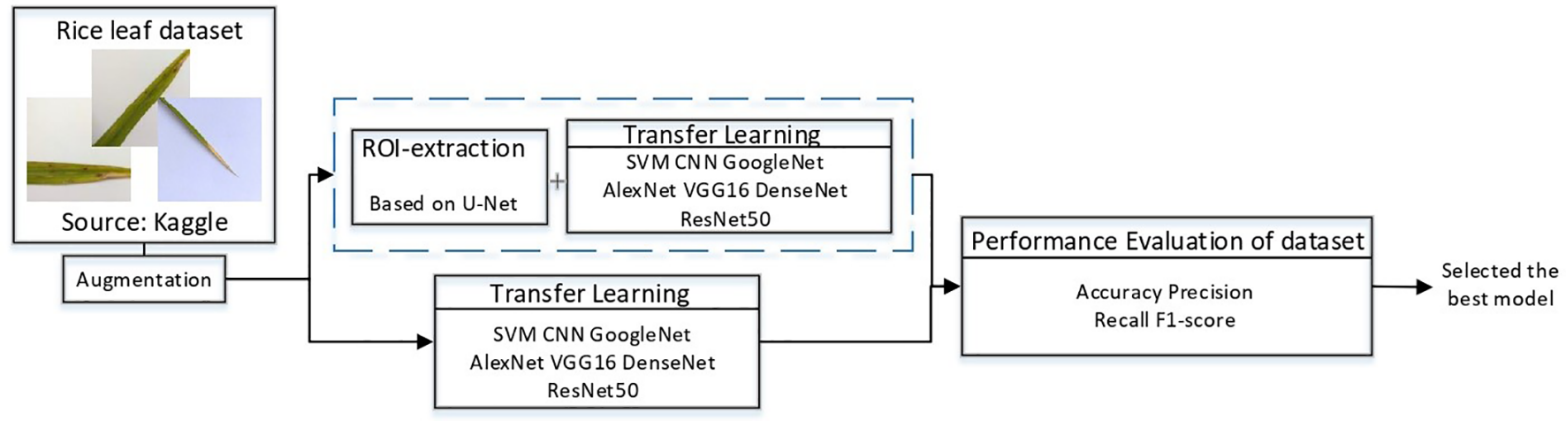
General block diagram of this study.

**Figure 5:**
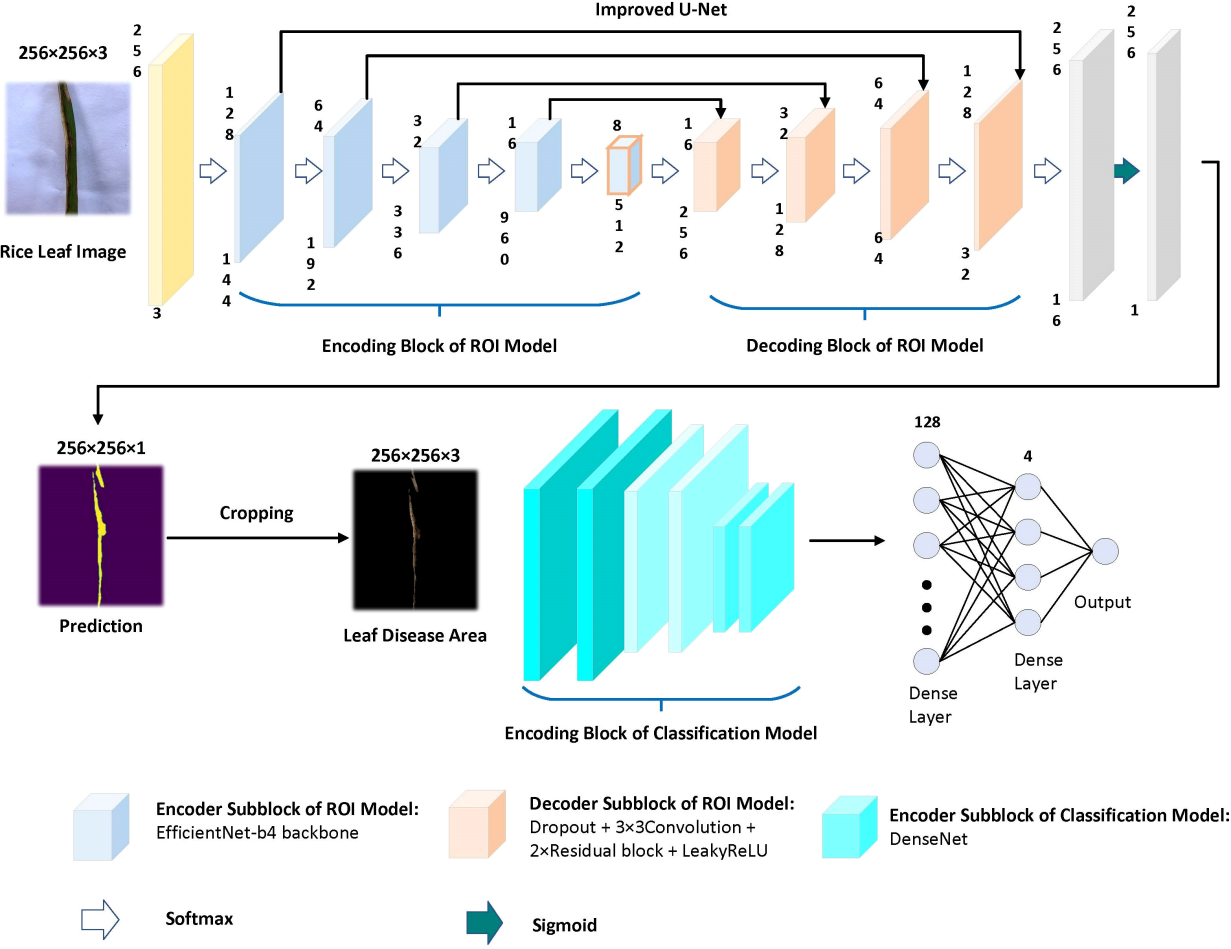
Overview of the recognition model. The upper part is the ROI extraction model, and the lower part is the DenseNet classification model.

In this study, a deep learning model for rice disease recognition was proposed, as shown in **Figure 4**. The rice leaf disease identification model proposed in this study consists of two parts: (1) ROI extraction model: input the rice leaf images, then locate and output the disease area pictures. (2) Classification sub-model: input the segmented disease areas and output the exact disease types. Therefore, the model proposed in this study contains two outputs, one is the segmented image of rice leaf disease parts obtained after ROI extraction, and the other is the rice leaf disease species output from the classification sub-model. The general framework of the model is shown in **Figure 5**.

### ROI extraction model

In image segmentation, particularly in the field of medical image segmentation, the U-Net model has performed extremely well (Siddique et al., 2021). The aim of using U-Net is to create pixel-level masks for each object in the images. The result is the identification and identification of the position and the shape of different objects in the images, classifying each pixel in each image into lesioned and non-lesioned areas (Ghosh et al., 2021). U-Net uses jump connections at the same stage (Ronneberger et al., 2015), rather than direct monitoring and loss-back transmission of high-level semantic features, thus ensuring that the final recovered feature map integrates more of the underlying features and also enables the fusion of features at different scales, thus allowing for multi-scale prediction and deep supervision. Up-sampling also enables finer information such as the recovered edges of segmented images. In the medical field and with a small number of samples, U-Net is still able to perform the image segmentation task excellently (Du et al., 2020). Medical image segmentation has some similarities with crop disease image segmentation.

Based on this, the improved U-Net model was chosen as the framework for the automatic segmentation model of leaf disease areas in this study. After several experiments, it was found that the improved U-Net combined with the Efficientnet-b4 pre-training network (based on the ImageNet dataset) performed the best in segmentation training.

**Figure 6:**
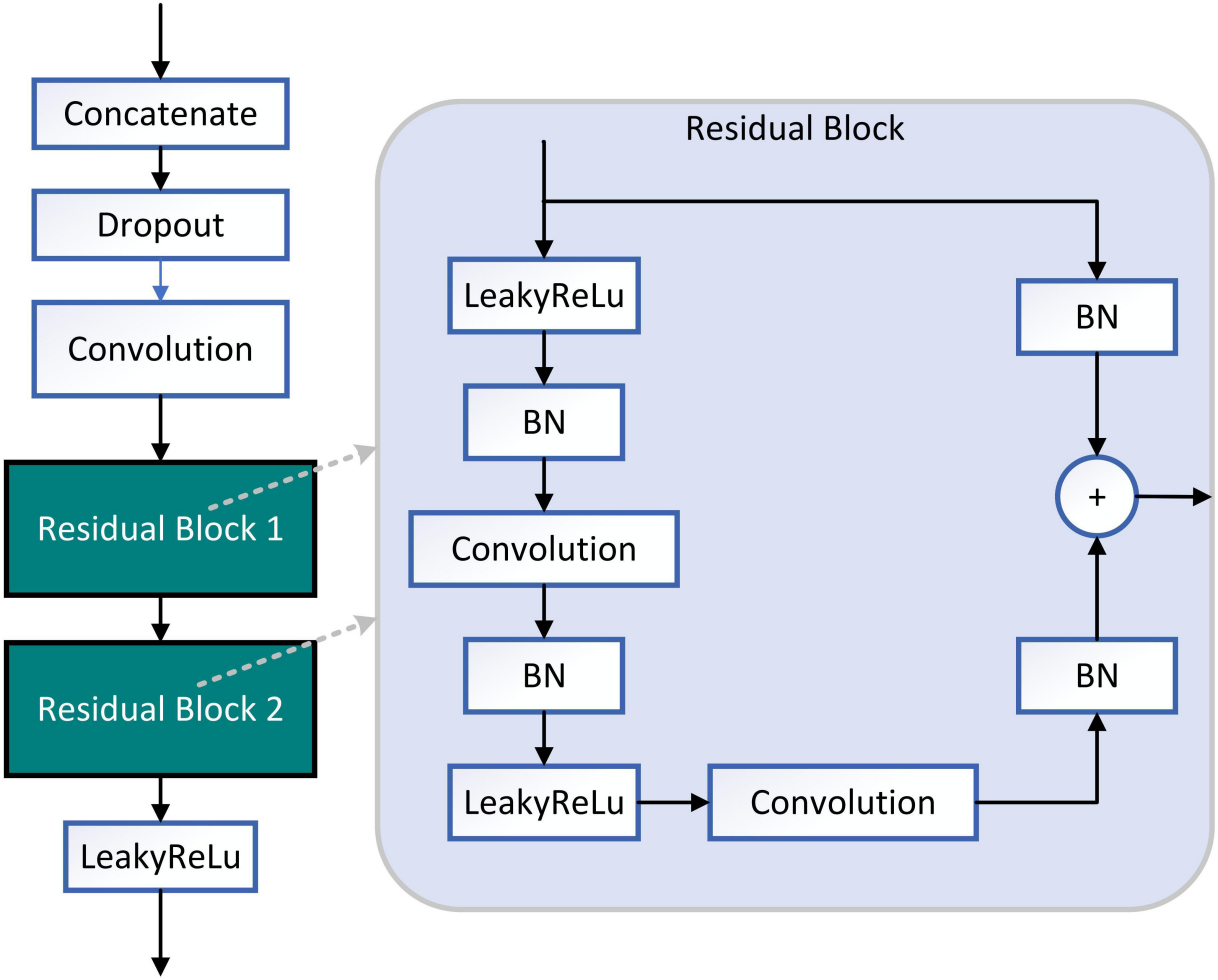
Structure diagram of decoding subblock. Two residual blocks are used in decoding block. The activate function of decoding subblock is LeakyReLU.

Using the pre-training Efficientnet-b4 as the encoder and the Residual block and LeakyRelu activation function in the decoder to improve the U-Net network (Liu et al., 2022). The encoder used in this study consists of Efficientnet-b4, and the decoder consists of five decoding subblocks, each of which includes a dropout layer, a 2D convolutional layer, a padding layer, two residual blocks, and a LeakyReLU layer, as shown in **Figure 6**. The dropout layer can effectively prevent model overfitting when the model has many parameters and few training samples. The LeakyReLU layer is used to improve the generalization ability of the model and prevent overfitting, and LeakyReLU is also used as the activation function of the middle layer to avoid neuron death. The two residual blocks prevent gradient vanishing and allow better information propagation. In addition, it was found during the experiments that using two residual blocks in series could yield higher segmentation accuracy. Finally, a 1×1 convolutional layer is applied and the ‘Sigmoid’ activation function (**Equation 1**) is used to output the mask.


(1) $$Sigmoid\left(x\right)=\frac{1}{1+e^{x}}$$

Where x represents the input of the function.

**Figure 7:**
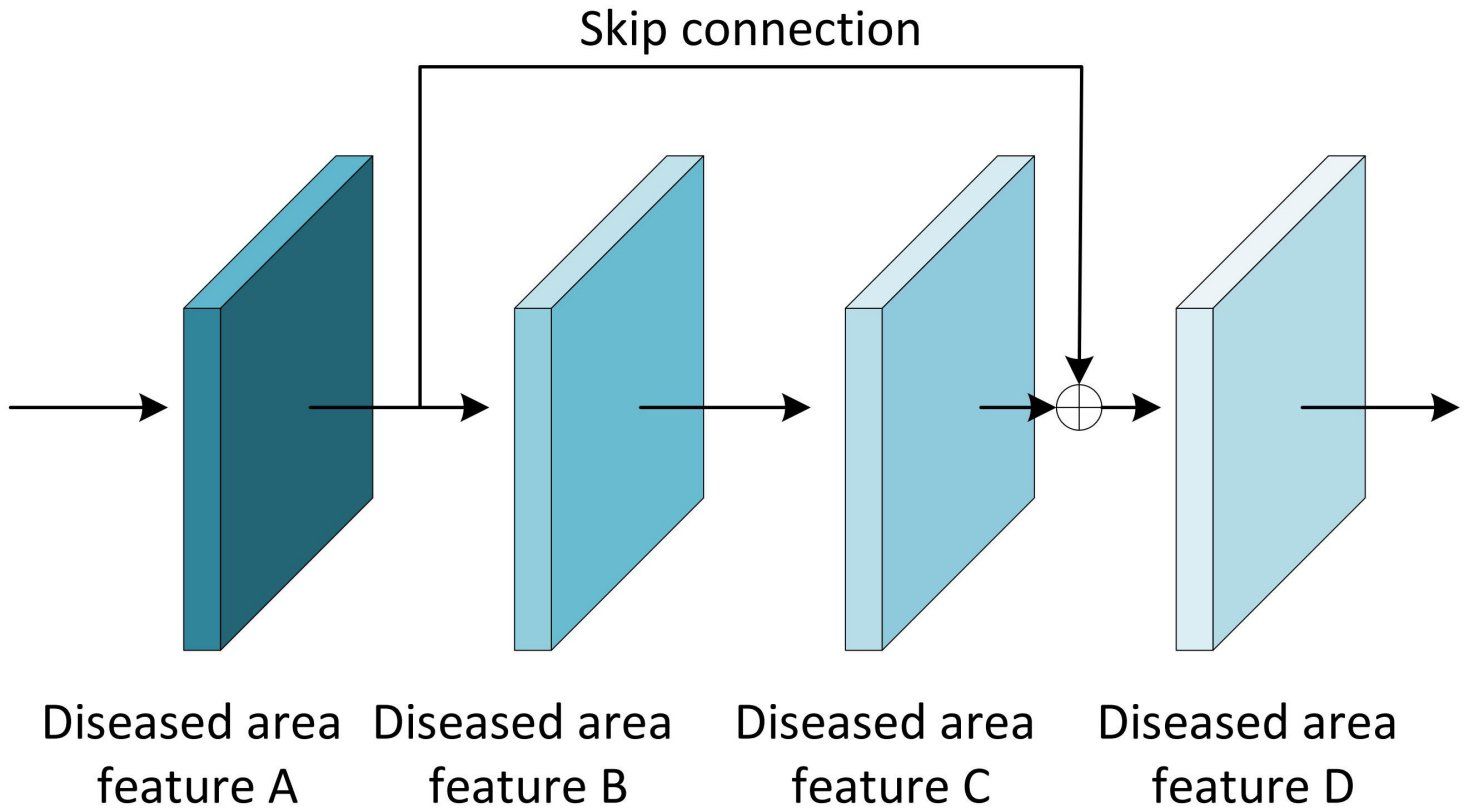
The principle of the residual block.

The Residual block is the most important module in the ResNet network. It adds a shortcut between the input and output of the network layer (He et al., 2016). The paradox is that while shallow networks do not improve performance significantly, deeper networks have a more pronounced ‘gradient vanishing’, which limits the effectiveness of training the network. However, the shortcut of residual block effectively solves the problem of ‘gradient vanishing’ when deepening the network, as shown in **Figure 7**. Even if the gradient decay occurs in the back-propagation of A-B-C, the gradient at D can still be transmitted directly to A. The gradient can be propagated across layers. In terms of gradient size, the residual network maintains a large weight value close to the data layer (input) to mitigate gradient vanishing, no matter how deep the network structure is.

LeakyReLU is often used as an activation function and works similarly to the ReLU function. The difference is that when the input is negative, the output of ReLU is 0, while the output of LeakyReLU is negative and with a gradient (Liu et al., 2019). Using ReLU as an intermediate layer activation function during backpropagation, the neurons will not update their weights and biases when the gradient is 0. Improved U-Net networks use LeakyReLU as an intermediate layer activation function to ensure computational speed, alleviate overfitting and also avoid neuron death.

### Disease classification sub-model

In this study, various pre-training models (based on the ImageNet dataset) were tried as tools for extracting image features, and finally, DenseNet with the highest classification accuracy was selected as the pre-training model. The experiments not only employed several popular neural network models including simple CNN, ResNet50, GoogleNet, VGG16, AlexNet, and DenseNet but also experimented with the machine learning method SVM. Each classification model is fine-tuned for training on this dataset. The total parameters used for each of the classification sub-models are shown in **Table 4**.

**Table 4:** TL models total parameters.

Models	Total parameter
SVM	32,534,628
Simple CNN	365,260
AlexNet	87,660,292
GoogleNet	23,905,060
ResNet50	157,814,660
VGG16	40,414,020
DensNet	18,568,388

**Figure 8:**
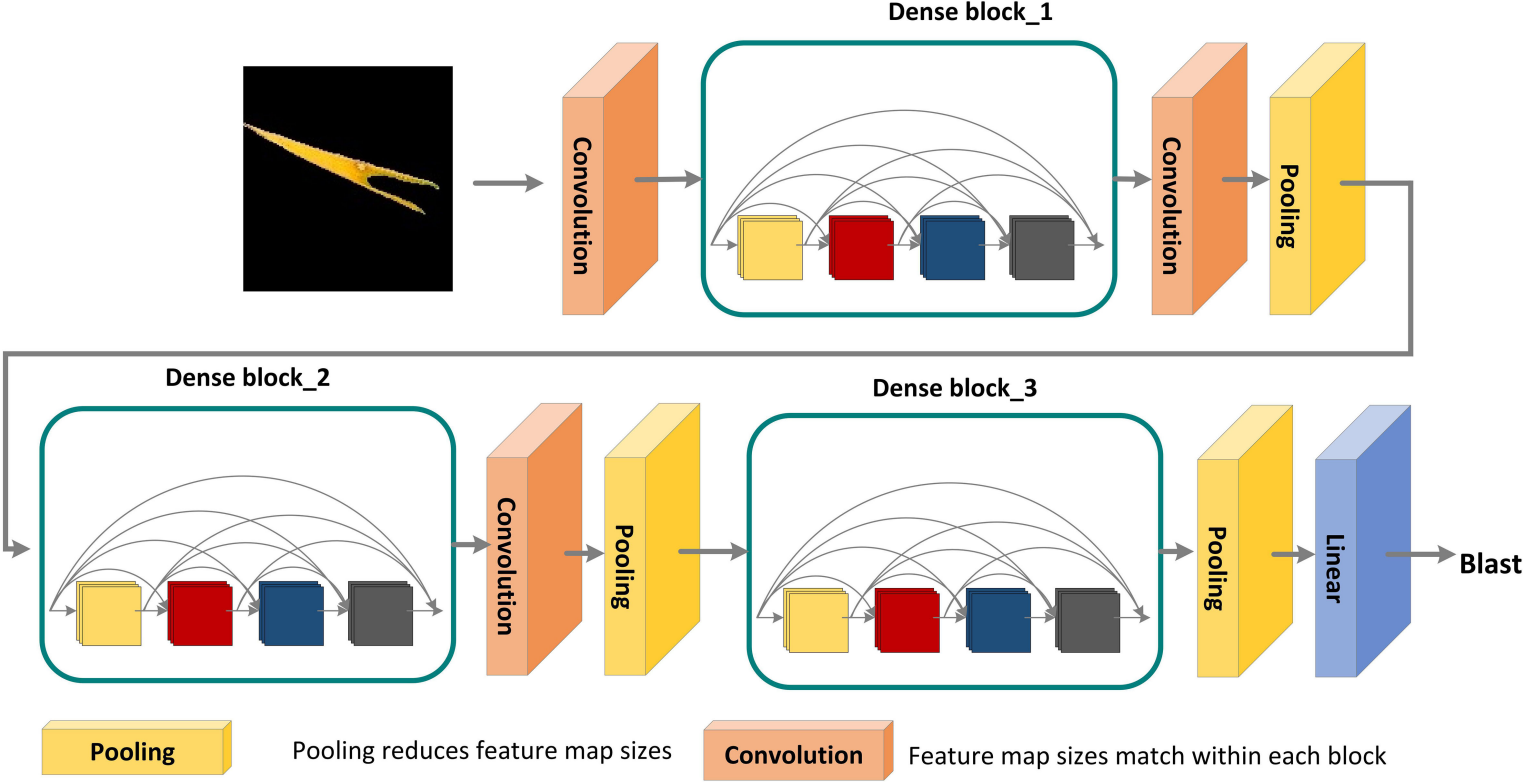
DenseNet pre-training model for disease areas classification. A deep DenseNet with three dense blocks. The layers between two adjacent blocks are referred to as transition layers and change feature-map sizes *via* convolution and pooling.

Each layer in the DenseNet model accepts all previous layers as its additional inputs, which is called the dense connectivity mechanism (Iandola et al., 2014). This can achieve feature reuse and reduce the number of parameters and computational cost of the network. To ensure that the size of the feature map is consistent across the layers (Huang et al., 2017), a dense block + transition structure is used in the DenseNet network, with the transition layer varying the feature map size through convolution and pooling, as shown in **Figure 8**. The transition layers used in the experiments consist of a batch normalization layer and a 1×1 convolutional layer followed by a 2×2 average pooling layer. The output from the DenseNet network is then flattened using the flatten layer, and the image information is parsed through two fully-connected layers with the ‘ReLU’ activation function (Equation 2) and the ‘Softmax’ activation function (Equation 3) to produce the classification results. The number of neurons in the final dense layer is 4 (indicating four classes). The exact network configurations are shown in **Table 5**.


(2) f(x)=max(0,x)

**Table 5:** DenseNet architectures.

Layers	Output size	DenseNet
Convolution	128×128	7 × 7 conv, stride 2
Pooling	64×64	3 × 3 max pool, stride 2
Dense Block (1)	64×64	$$\left(\begin{array}{c} 1\times 1\;\text{conv}\\ 3\times 3\;\text{conv} \end{array}\right)\times 6$$
Transition Layer (1)	64×64	1×1 conv
	32×32	2×2 average pool, strid 2
Dense Block (2)	32×32	$$\left(\begin{array}{c} 1\times 1\;\text{conv}\\ 3\times 3\;\text{conv} \end{array}\right)\times 6$$
Transition Layer (2)	32×32	1×1 conv
	16×16	2×2 average pool, strid 2
Dense Block (3)	16×16	$$\left(\begin{array}{c} 1\times 1\text{ conv}\\ 3\times 3\text{ conv} \end{array}\right)\times 48$$
Transition Layer (3)	16×16	1×1 conv
	8×8	2×2 average pool, strid 2
Dense Block (4)	8×8	$$\left(\begin{array}{c} 1\times 1\text{ conv}\\ 3\times 3\text{ conv} \end{array}\right)\times 32$$
Classification Layer	1×1	7×7 global average pool
		128D full-connected ‘relu’
		4D full-connected ‘softmax’


(3) $$y_{i}=S{\left(z\right)}_{i}=\frac{e^{z_{i}}}{\sum_{j=1}^{C}e^{z_{j}}}\;,i=1,\ldots ,C$$

Where z represents the output of the last layer, and C represents the dimension.

### Metrics and hyper-parameters

The Dice (Equation 4) and Dice Loss (Equation 5) were chosen as the metrics for the split model, while the classification sub-model metrics were Accuracy (Equation 6), Precision (Equation 7), Recall (Equation 8), F1 (Equation 9) and AUC (Equation 10).


(4) $$Dice=\frac{2TP}{2TP+FN+FP}$$

(5) Dice Loss=1−Dice

(6) $$Accuracy=\frac{TP+TN}{TP+FP+TN+FN}$$

(7) $$Precision=\frac{TP}{TP+FP}$$

(8) $$Recall=\frac{TP}{TP+FN}$$

(9) $$F1=\frac{2TP}{2TP+FN+FP}$$

Where True Positive (TP) represents the number of correctly predicted positive samples, False Positive (FP) represents the number of incorrectly predicted positive samples, True Negative (TN) represents the number of correctly predicted negative samples, and False Negative (FN) represents the number of incorrectly predicted negative samples.


(10) $$AUC=\frac{\sum_{i\in positiveClass}rank_{i}-\frac{M\times \left(M+1\right)}{2}}{M\times N}$$

Where M represents the number of positive samples, and N represents the number of negative samples.

The initial learning rate of the model was set to 0.0002. The optimizer of the model was adam. The batch size was set to 2. Max epochs were set to 100. When the Dice of the model was not improved over five epochs, the learning rate will drop by 50 percent.

## Results

### ROI extraction model training results

**Figure 9:**
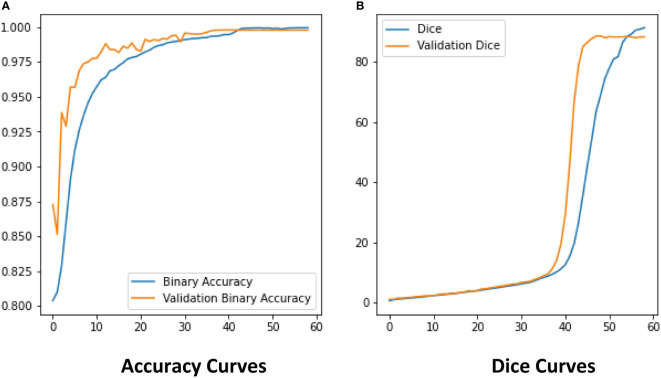
The Dice and Binary Accuracy of ROI model. **(A)** Accuracy Curves, **(B)** Dice Curves.

**Figure 10:**
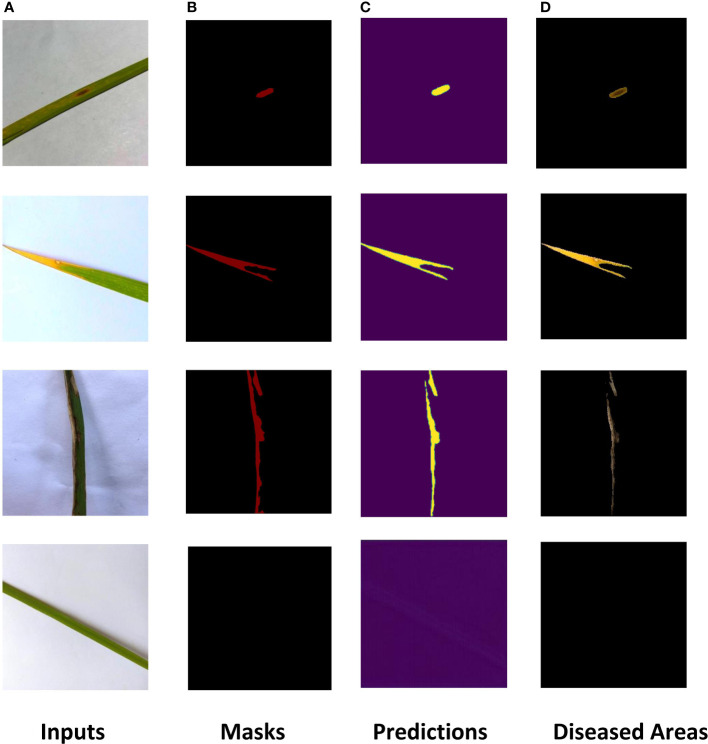
Results of ROI extraction. **(A)** Inputs, **(B)** Masks, **(C)** Predictions, **(D)** Diseased Areas.

The ROI extraction model under the improved U-Net framework was used to extract diseases of rice leaf images. In order to prevent the model from overfitting, the Early-stopping algorithm and the learning rate decay strategy ReduceLROnPlateau were used to continuously update its learning rate. After adjusting the parameters several times, the model achieved excellent segmentation results, and the optimal value of Dice of the extraction results reached 0.86 by the 5-fold cross-validation method. The Dice and Accuracy variation curves of the training process are shown in **Figure 9**. **Figure 10** shows the ROI extraction results, where **(A)** are the original image inputs, **(B)** are the masks, **(C)** are the model predictions, and **(D)** are the segmented lesion areas.

### Classification sub-model training results

To testify the help of the ROI extraction algorithm on the identification accuracy of the classification model, images with and without ROI extraction were put into the training of the classification sub-model to obtain the classification accuracy of leaves. Considering the size of the dataset, the experiment uses Bootstrap Method to enhance the classification effect. **Tables 6**, **7** show the disease classification results of rice leaf images without and with ROI extraction, respectively. The experiments not only employed several popular neural network models including simple CNN, ResNet50, GoogleNet, VGG16, AlexNet, and DenseNet but also experimented with the machine learning method SVM.

**Table 6:** Classification results without ROI extraction.

Models	Types	Precision	Recall	F1-score	Accuracy
SVM	Leaf Blast	0.90	0.97	0.93	0.82
	Bacterial Blight	0.65	0.78	0.71	
	Brown spot	0.23	0.12	0.16	
	Healthy	0.54	0.33	0.41	
Simple CNN	Leaf Blast	0.91	0.94	0.92	0.81
	Bacterial Blight	0.65	0.67	0.66	
	Brown spot	0.26	0.24	0.22	
	Healthy	0.56	0.48	0.52	
AlexNet	Leaf Blast	0.92	0.92	0.92	0.78
	Bacterial Blight	0.54	0.61	0.57	
	Brown spot	0.31	0.16	0.21	
	Healthy	0.45	0.66	0.54	
GoogleNet(InceptionV3)	Leaf Blast	0.91	0.95	0.93	0.86
	Bacterial Blight	0.86	0.82	0.84	
	Brown spot	0.50	0.38	0.43	
	Healthy	0.64	0.52	0.57	
ResNet50	Leaf Blast	0.12	0.37	0.18	0.59
	Bacterial Blight	0.22	0.21	0.21	
	Brown spot	0.16	0.16	0.16	
	Healthy	0.63	0.76	0.69	
VGG16	Leaf Blast	0.90	0.92	0.91	0.78
	Bacterial Blight	0.51	0.50	0.50	
	Brown spot	0.27	0.26	0.26	
	Healthy	0.46	0.43	0.44	
DenseNet	Leaf Blast	0.82	0.84	0.83	0.86
	Bacterial Blight	0.72	0.90	0.80	
	Brown spot	0.64	0.30	0.41	
	Healthy	0.72	0.75	0.73	

**Table 7:** Classification results with ROI extraction.

**Models**	**Types**	**Precision**	**Recall**	**F1-score**	**Accuracy**
ROI+SVM	Leaf Blast	0.61	0.97	0.66	0.92
	Bacterial Blight	0.98	1.00	0.99	
	Brown spot	0.98	0.91	0.94	
	Healthy	0.98	0.90	0.93	
ROI+Simple CNN	Leaf Blast	0.73	0.83	0.78	0.88
	Bacterial Blight	0.96	0.96	0.96	
	Brown spot	0.86	0.73	0.79	
	Healthy	0.92	0.92	0.92	
ROI+AlexNet	Leaf Blast	0.74	0.69	0.71	0.85
	Bacterial Blight	0.95	0.98	0.96	
	Brown spot	0.79	0.72	0.75	
	Healthy	0.88	0.92	0.90	
ROI+GoogleNet(InceptionV3)	Leaf Blast	0.66	0.95	0.78	0.92
	Bacterial Blight	0.97	0.90	0.93	
	Brown spot	0.98	0.87	0.92	
	Healthy	0.97	0.94	0.95	
ROI+ResNet50	Leaf Blast	0.67	0.38	0.49	0.77
	Bacterial Blight	0.79	0.87	0.83	
	Brown spot	0.76	0.50	0.60	
	Healthy	0.79	0.95	0.86	
ROI+VGG16	Leaf Blast	0.75	0.70	0.72	0.86
	Bacterial Blight	0.89	0.71	0.79	
	Brown spot	0.83	0.91	0.92	
	Healthy	0.87	0.83	0.85	
ROI+DenseNet	Leaf Blast	0.87	0.77	0.82	0.96
	Bacterial Blight	0.97	0.94	0.95	
	Brown spot	0.98	0.94	0.96	
	Healthy	0.97	0.99	0.98	

**Figure 11:**
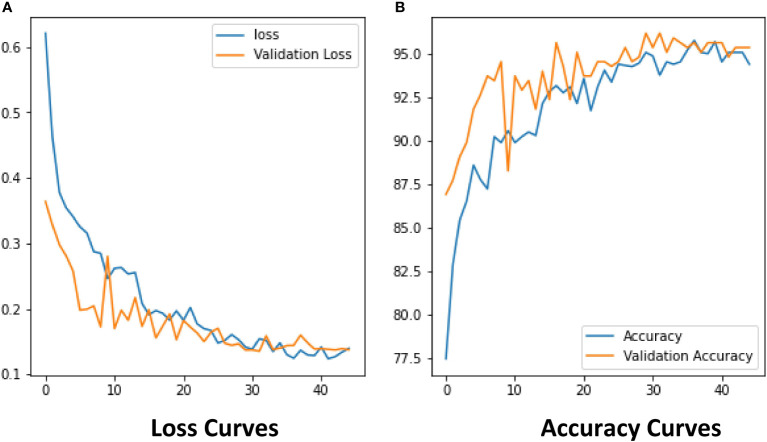
The Loss and Accuracy of classification model. **(A)** Loss Curves, **(B)** Accuracy Curves.

**Figure 12:**
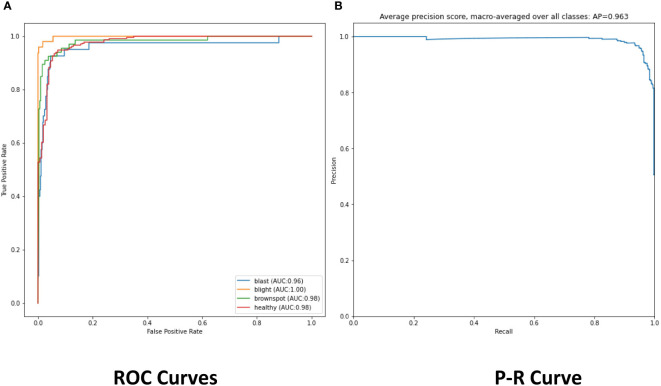
**(A)** The ROC of classification model. **(B)** The Precision and Recall of classification model.

**Figure 13:**
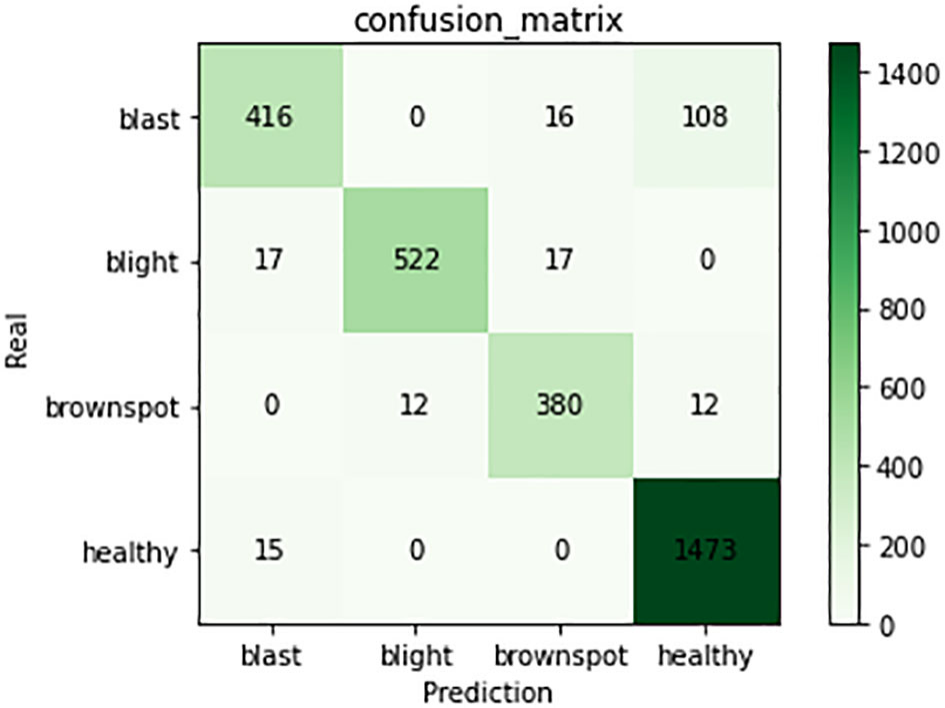
Confusion matrix of classification model. The figure shows the number of predictions for four types of training samples.

The experimental results show that the DenseNet model performs significantly better in the classification task than the other neural network models in the experiment. The single DenseNet classification model was able to achieve an average disease identification accuracy of 86% by the 5-fold cross-validation method. In contrast, the ROI feature extraction algorithm combined with the DenseNet model achieved identification accuracy of 96% by the 5-fold cross-validation method, an improvement of 10% over the single classification model. The accuracy and loss curves during the training of the DenseNet classification model combined with the ROI extraction algorithm are shown in **Figure 11**, and **Figure 12** shows the identification model classification ROC and P-R curves. The confusion matrix of classification results is shown in **Figure 13**.

A comparison between this study and existing related studies is shown in **Table 8**. Classification of paddy leaf diseases using an optimized deep neural network with the Jaya algorithm is proposed. This method achieved high accuracy of 98.9% for the blast affected, 95.78% for the bacterial blight, 92% for the sheath rot, 94% for the brown spot, and 90.57% for the normal leaf image (Ramesh and Vydeki, 2020). Li et al. (2020) used faster-RCNN to detect rice videos. The proposed method was more suitable for the detection of the rice videos than VGG16, ResNet, and YOLOv3. Prajapati et al. (2017) used SVM for multi-class classification and achieved 93.33% accuracy on the training dataset and 73.33% accuracy on the test dataset. Rahman et al. (2020) proposed simple-CNN model can achieve the desired accuracy of 93.3% with a significantly reduced model size. N. Krishnamoorthy and Parameswari (2021) used transfer learning to identify leaf blast, bacterial blight, and brown spot. The classification accuracies for the VGG-16, ResNet50, and InceptionV3 CNN models were 87%, 93%and 95% respectively. Deng et al. (2021) used the ensemble model to diagnose six types of rice diseases, and overall accuracy of 91% was achieved. Their dataset contained 33026 images of six types of rice diseases: leaf blast, false smut, neck blast, sheath blight, bacterial stripe disease, and brown spot. The experimental results of this study were not inferior to other studies and accurate identification of rice leaf diseases could be accomplished.

**Table 8:** Comparison of existing related studies.

Researchers	Method	Number of observation	Performance (%)
Ramesh & Vydeki (2020)	Optimized Deep Neural Network with Jaya Optimization Algorithm (DNN_JOA)	650	Rice blast: 98.9 Bacterial blight: 95.78 Sheath rot: 92 Brown spot: 94 Normal leaf: 90.57
Li et al. (2020)	Faster-RCNN	5320	Rice sheath blight:90.9 Rice stem borer: 71.4 Rice brown spot: 90
Prajapati et al. (2017)	SVM	120	For SVM: 93.33 (training) 73.33 (testing)
Rahman et al. (2020)	Simple CNN	1,426	Mean validation accuracy: 94.33
Krishnamoorthy and Parameswari (2021)	InceptionV3	1000	Mean validation accuracy:95.41
Deng et al. (2021)	Deep Learning	33026	Accuracy:91
Proposed Model	DenseNet model with ROI extraction algorithm	2988	Mean validation accuracy:96

## Discussion

In this study, a hybrid DenseNet model based on improved U-Net is proposed to replace manual identification which is time-consuming, inefficient, and poorly coordinated. In which the improved U-Net is used for ROI extraction and the DenseNet is used for image classification. In order to select the most suitable classification network, several popular neural network models, including Simple CNN, SVM, ResNet50, GoogleNet, VGG16, AlexNet, and DenseNet, have been subjected to classification experiments of images with and without ROI extraction. In the classification experiments of images without ROI extraction, the accuracy of Simple CNN is 81%, the accuracy of SVM is 82%, the accuracy of ResNet50 is 59%, the accuracy of GoogleNet is 86%, the accuracy of VGG16 is 78%, the accuracy of AlexNet is 78%, and the accuracy of DenseNet is 0.86 In the hybrid ROI extraction classification experiment, the optimal value of Dice for ROI extraction results reached 0.86. In the experiments with ROI extraction processed images, the accuracy of Simple CNN is 88%, SVM is 92%, ResNet50 is 77%, GoogleNet is 0.86, VGG16 is 86%, AlexNet is 85%, and DenseNet is 96%. Combining the results of two experiments, the ROI extraction based on the improved U-Net improves the classification performance of Simple CNN, SVM, ResNet50, GoogleNet, VGG16, AlexNet, and DenseNet on the dataset of this study, with DenseNet performing the best. Due to the difficulty in collecting original training samples, the dataset used in the present study only included images of three rice leaf diseases. The original training samples used in the study were taken under well-lit environmental conditions, which may limit the scenarios for use in practical applications. In addition, only seven neural network models were considered in this study. Although these models met our experimental needs, the possibility that other neural network models will yield better results cannot be ruled out. These issues are yet to be addressed in future studies.

## Conclusion

This study is based on improved U-Net and uses DenseNet to diagnose rice leaf diseases, which automatically locates and extracts rice leaf disease areas and identifies three common rice leaf diseases (brown spot, leaf blast, and bacterial blight). The segmentation dice coefficient can reach 0.86 by extracting the lesion area through the improved U-Net. Among the tested seven neural network models, including Simple CNN, SVM, ResNet50, GoogleNet, VGG16, AlexNet, and DenseNet, DenseNet performed the best in the training of disease classification for lesion area images with an accuracy of 96%. Improving U-Net improved the recognition accuracy of the seven neural network models, with DenseNet’s accuracy increasing by 10%. The disease area pictures obtained by the segmentation model can be used to assist in localization and aid in identification, which is beneficial to modern farm management, and the classification model can help laymen to identify disease species. It is convenient, real-time, and practical to assist disease classification through ROI extraction. Managers can observe the condition of rice fields through drones. In future works, we intend to add more types of rice leaf disease images to dataset and train them allowing this method to accomplish more rice disease identification. In addition, we intend to add images of the original training samples under different environmental conditions may improve the reliability of the model. We also intend to consider different crops as new research targets, e.g. tomato, wheat, maize, etc., to extend the applicability of this study to achieve disease identification and control of multiple crops.

## Data availability statement

The raw data supporting the conclusions of this article will be made available by the authors, without undue reservation.

## Author contributions

WL and LY wrote the main manuscript text. WL, LY, and JL performed experiments and prepared figures. LY cleansed the dataset. LY prepared the dataset and confirmed abnormalities. All authors reviewed the manuscript. All authors contributed to the article and approved the submitted version.

## Funding

This work was supported by the Henan university of technology (No.2017RCJH12 & No.2018008).

## Conflict of interest

The authors declare that the research was conducted in the absence of any commercial or financial relationships that could be construed as a potential conflict of interest.

## Publisher’s note

All claims expressed in this article are solely those of the authors and do not necessarily represent those of their affiliated organizations, or those of the publisher, the editors and the reviewers. Any product that may be evaluated in this article, or claim that may be made by its manufacturer, is not guaranteed or endorsed by the publisher.
